# Antifungal Activity of a Neodymium-Doped Yttrium Aluminum Garnet 1,064-Nanometer Laser against Sporothrix globosa by Inducing Apoptosis and Pyroptosis via the NLRP3/Caspase-1 Signaling Pathway: *In Vitro* and *In Vivo* Study

**DOI:** 10.1128/Spectrum.01364-21

**Published:** 2021-12-15

**Authors:** Tianyi Yan, Fuqiu Li, Jinran Li, Feng Chen

**Affiliations:** a Department of Dermatology, Second Hospital of Jilin University, Changchun, Jilin, China; b Department of Dermatology, China-Japan Union Hospital of Jilin University, Changchun, Jilin, China; Institut Pasteur

**Keywords:** NLRP3, Nd:YAG, 1,064-nm laser, *Sporothrix globosa*, Th1 and Th17 cell response, apoptosis, pyroptosis

## Abstract

Sporotrichosis is a deep fungal infection caused by *Sporothrix* species. Currently, itraconazole is the main treatment, but fungal resistance, adverse effects, and drug interactions remain major concerns, especially in patients with immune dysfunction. Therefore, an alternative treatment is greatly in demand. This animal study aimed to investigate the inhibitory effect of neodymium-doped yttrium aluminum garnet (Nd:YAG) 1,064-nm laser treatment on Sporothrix globosa and to explore whether it happens through regulation of the Nod-like receptor thermoprotein domain-related protein 3 (NLRP3)/caspase-1 pyroptosis and apoptosis pathway. After laser irradiation, a series of studies, including assays of viability (using the cell counting kit-8 [CCK-8]), morphological structure changes, reactive oxygen species (ROS) accumulation, mitochondrial membrane potential, oxidative stress, cell cycle progression, and metacaspase activation, were conducted to estimate the effect of Nd:YAG 1,064-nm laser treatment on Sporothrix globosa cell apoptosis *in vitro*. For *in vivo* studies, mice were infected with S. globosa and then treated with laser or itraconazole, and their footpad skin lesions and the changes in the histology of tissue samples were compared. In addition, changes in the levels of NLRP3, caspase-1, and caspase-3 were assessed by immunohistochemistry, while the levels of interleukin 17 (IL-17), interferon gamma (IFN-γ), and transforming growth factor β1 (TGF-β1) in peripheral blood were tested by enzyme-linked immunosorbent assay (ELISA). The *in vitro* growth of S. globosa was inhibited and apoptosis was observed after laser treatment. According to the *in vivo* studies, the efficacy of the laser treatment was similar to that of itraconazole. Moreover, the NLRP3/caspase-1 pyroptosis pathway was activated, with a Th1/Th17 cell response, and the expression of caspase-3 was also upregulated. Nd:YAG 1,064-nm laser treatment can effectively inhibit the growth of S. globosa by activating fungal apoptosis and pyroptosis through the NLRP3/caspase-1 pathway. Therefore, Nd:YAG 1,064-nm laser irradiation is an alternative for sporotrichosis therapy.

**IMPORTANCE** Nd:YAG 1,064-nm laser irradiation is a useful alternative for the treatment of sporotrichosis, especially in patients with liver dysfunction, pregnant women, and children, for whom the administration of antifungal drugs is not suitable. It may improve the overall treatment effect by shortening the duration of antifungal treatment and reducing tissue inflammation.

## INTRODUCTION

Sporotrichosis is a deep fungal infection caused by *Sporothrix* species (1). Sporothrix globosa is the most common pathogenic species of *Sporothrix* in Northeast China (2, 3), causing localized and even severe systemic disseminated infections. Currently, itraconazole is the main treatment, but fungal resistance, adverse effects, and drug interactions remain major concerns, especially in patients who have immune dysfunction (4–6). Therefore, an alternative treatment is greatly in demand.

Geusic et al. (7) first demonstrated the neodymium-doped yttrium aluminum garnet (Nd:YAG) laser at the Bell Laboratories in 1964. The Nd:YAG laser has a wavelength of 1,064 nm and is capable of reaching deeper layers of tissue than other types of lasers (8). It has the advantage of being able to treat deep fungal infections. In recent years, the Nd:YAG laser has mostly been used to treat onychomycoses (9), Candida albicans (10), chromomycosis (11), warts (12), and some vascular and pigmentary diseases (13). In our early clinical study, we found that utilizing the Nd:YAG 1,064-nm laser as an additional treatment shortened the duration of sporotrichosis therapy, reduced the amount of azole antifungal drugs used, and decreased drug resistance. Moreover, Nd:YAG 1,064-nm laser treatment did not aggravate residual pigments, scars, pain, or recurrence of lesions (14). However, the specific inhibitory effect on sporotrichosis is still unclear (15, 16).

Several recent studies have indicated that an imbalance of CD4^+^ T cell subsets and dysregulation of cytokines are closely correlated with host resistance to sporotrichosis (17, 18). The Nod-like receptor thermoprotein domain-related protein 3 (NLRP3)/caspase-1 pyroptosis pathway plays an important role in this. Gonçalves et al. (19) suggested that the NLRP3 inflammasome limited the progression of sporotrichosis by regulating the adaptive immunity mediated by CD4^+^ T cell subsets. NLRP3 and caspase-1 knockout mice were more susceptible to sporotrichosis. The proportions of Th17 cells and Th1/Th17 cells in gene knockout mice infected with sporotrichosis were decreased, suggesting that the NLRP3 inflammasome has antisporotrichosis immune activity. We found an imbalance of Th1, Th17, and regulatory T (Treg) cells in the peripheral blood of patients with sporotrichosis. The levels of the cells were associated with the duration of the disease (20). However, no study has found that these cells are associated with the NLRP3/caspase-1 pyroptosis pathway or that they are activated by laser treatment. Indeed, the regulatory effects of the NLRP3/caspase-1 pyroptosis pathway against sporotrichosis upon Nd:YAG 1,064-nm laser treatment are worthy of further study. Additionally, caspase-3 is a critical mediator of apoptosis, and NLRP3 is also associated with apoptosis (21, 22).

This animal study (level of evidence: therapeutic study, level Ia) aimed to investigate the inhibitory effect of Nd:YAG 1,064-nm laser treatment on S. globosa and to explore whether it happens through regulation of the NLRP3/caspase-1 pyroptosis and apoptosis pathway. In addition, we compared the efficiency of laser treatment with that of itraconazole for the treatment of sporotrichosis.

## RESULTS

### Optimal laser energy.

**FIG 1:**
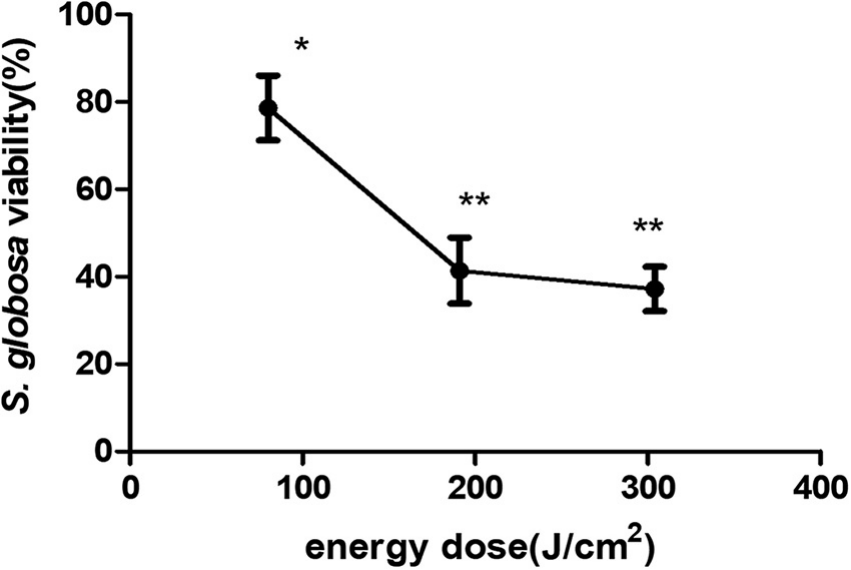
CCK-8 viability assay showing the inhibiting effects of different laser energies on Sporothrix globosa cells *in vitro*. *, mean value compared to HC group, *P* < 0.05; **, mean value compared to HC group, *P* < 0.01.

We identified the optimal laser energy against S. globosa as 400 J/cm^2^ (the actual energy density absorbed by S. globosa cells was 191 J/cm^2^). The results showed that the proportion of viable S. globosa cells decreased significantly after treatment with laser energy at 400 J/cm^2^ (actual absorption, 191 J/cm^2^), a greater effect than was seen for 200 J/cm^2^ (the actual energy density absorbed by the S. globosa cells was 80 J/cm^2^). There was no significant difference between the results using 400 J/cm^2^ and 600 J/cm^2^ (the actual energy density absorbed by the S. globosa cells was 304 J/cm^2^) (Fig. 1).

### Morphological changes of S. globosa cells after laser irradiation.

**FIG 2:**
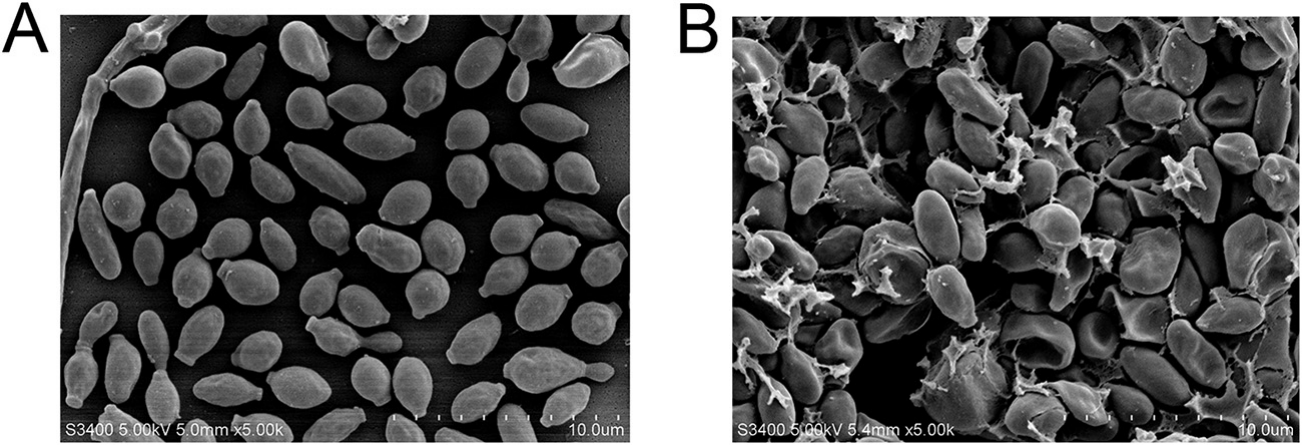
(A) Under a scanning electron microscope, the normal morphology of S. globosa cells is oval shaped and plump. (B) After laser irradiation, the S. globosa cells are damaged and shriveled.

The normal morphology of S. globosa cells was observed in the control group, which showed regular oval shapes, complete and smooth cell surfaces, and clearly visible boundaries (Fig. 2A). In the laser irradiation group, the morphological changes of S. globosa cells included shrunken membranes, leakage of contents, and cross-linking (Fig. 2B).

### Increased S. globosa cell apoptosis and necrosis after laser irradiation.

**FIG 3:**
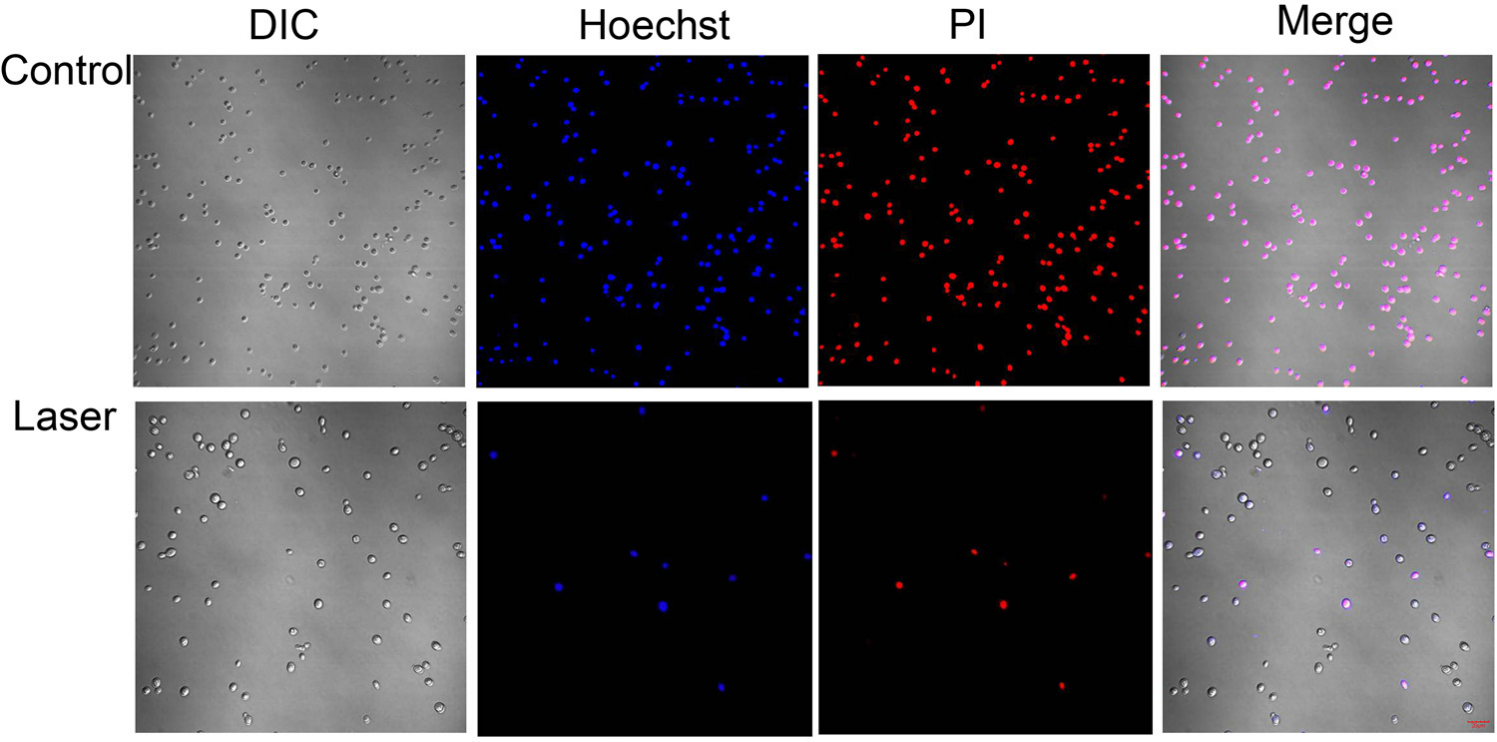
Confocal laser scanning microscopy was used to evaluate the effects of laser treatment on S. globosa cell necrosis and apoptosis as observed by Hoechst/propidium iodide staining.

Normal S. globosa cells showed weak red and blue fluorescence. Apoptotic S. globosa cells showed weak red and strong blue fluorescence. Necrotic S. globosa cells showed strong red and blue fluorescence. We observed that the laser exposure increased the Hoechst and propidium iodide staining signals, indicating a high presence of apoptotic and necrotic cells (Fig. 3).

### Increased S. globosa oxidative stress sensitivity after laser irradiation.

**FIG 4:**
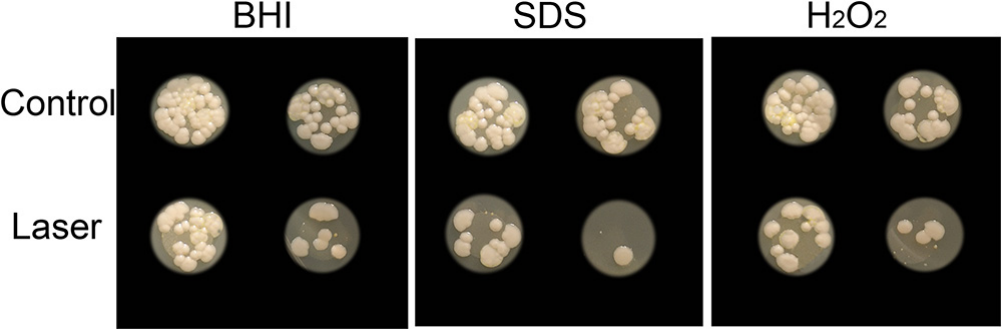
Laser treatment affected the oxidative stress sensitivity of S. globosa cells. S. globosa cells of the control and study groups were plated on brain heart infusion (BHI), SDS, and H_2_O_2_ media.

Laser exposure increased the susceptibility of S. globosa cells to oxidative stress inductance. Laser irradiation increased the sensitivity of S. globosa cells to H_2_O_2_ or SDS, resulting in inhibition of the growth of S. globosa (Fig. 4).

### Mitochondrial membrane potential change and ROS accumulation of S. globosa cells after laser irradiation.

**FIG 5:**
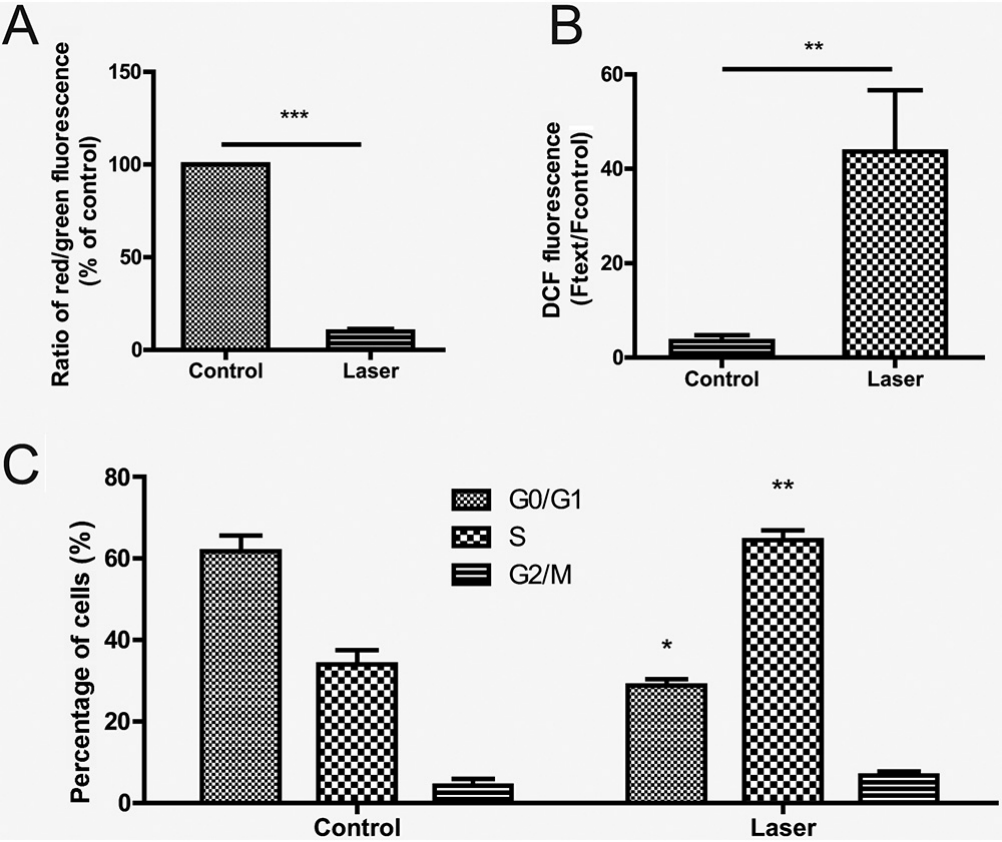
Measurement of mitochondrial membrane potential, intracellular reactive oxygen species (ROS) accumulation, and cell cycle progression of S. globosa cells after being treated with the laser. (A) S. globosa cells of the study and control groups were incubated with JC-1 dye, and the mitochondrial membrane potential was measured. (B) After staining with DCFH-DA, the ROS levels of S. globosa cells were measured. (C) S. globosa cells were stained with propidium iodide, and the percentages of stained cells in different cell cycle phases were analyzed. The results are the average values ± standard deviations from three independent experiments. Ftext, fluorescence value of the sample after laser treatment. *, mean value compared to control group, *P* < 0.05; **, mean value compared to control group, *P* < 0.01; ***, mean value compared to control group, *P* < 0.001.

The fluorescence ratio of S. globosa cells of the control group was almost 100%, which proved that there was no obvious depolarization. However, the fluorescence ratio of S. globosa cells in the laser irradiation group was reduced to 9.9% ± 1.4% (mean ± standard deviation) (*P* < 0.001) (Fig. 5A). After laser irradiation, the reactive oxygen species (ROS) accumulation of S. globosa cells was 43.7% ± 7.8%, a significant elevation above the accumulation in the control group (3.5% ± 1.3%) (*P* < 0.001) (Fig. 5B). The outcome indicated that laser-induced apoptosis was achieved through decreasing the mitochondrial activity and promoting ROS accumulation.

### Cell cycle block in S-phase S. globosa cells after laser irradiation.

After laser irradiation, the proportion of S-phase S. globosa cells reached 64.5% ± 4.9%, significantly higher than the proportion in the control group (28.8% ± 3.3%). These results indicated that the laser irradiation promoted blocking of the cell cycle in the S phase (*P* < 0.001), affecting DNA synthesis (Fig. 5C).

### Activation of S. globosa metacaspase after laser irradiation.

**FIG 6:**
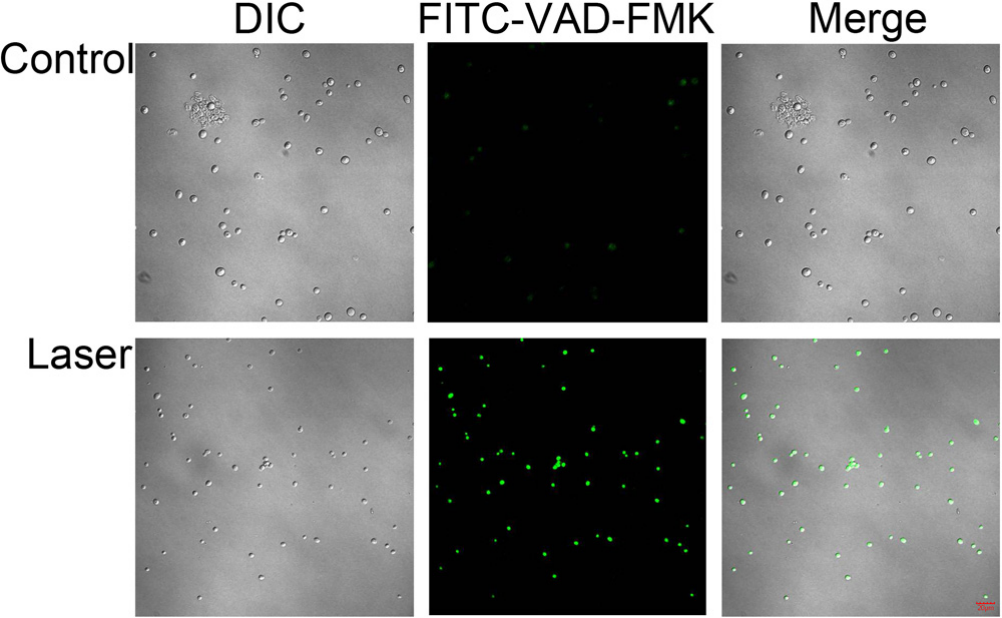
Confocal laser scanning microscopy to evaluate the effects of laser treatment on metacaspase activation in S. globosa cells of the study and control groups as observed by staining with CaspACE FITC-VAD-FMK. DIC, differential inference contrast.

The activation of metacaspase was detected by the CaspACE fluorescein isothiocyanate (FITC)-VAD-FMK *in situ* marker, and green fluorescence revealed activated intracellular metacaspase in S. globosa cells. We found that no fluorescence was observed for S. globosa cells in the control group, while in the laser irradiation group, most of the S. globosa cells were labeled with bright green fluorescence (Fig. 6), which indicated that the laser treatment activated the caspase-dependent apoptosis pathway in S. globosa.

### Size of laser-irradiated mouse footpads after S. globosa infection.

In the study group of mice, the footpads of the mice began to get red and swollen on day 2 after S. globosa inoculation. Subsequently, the swelling gradually increased, with ulceration, suppuration, and significant inflammatory lumps. On day 10, the S. globosa infection model had been built successfully, as confirmed by three histopathological tests (see Fig. 8B). After that, we divided the mice of the study group into an untreated-infection group (infection group; *n* = 18), a laser treatment group (*n* = 18), and an itraconazole treatment group (*n* = 18).

**FIG 7:**
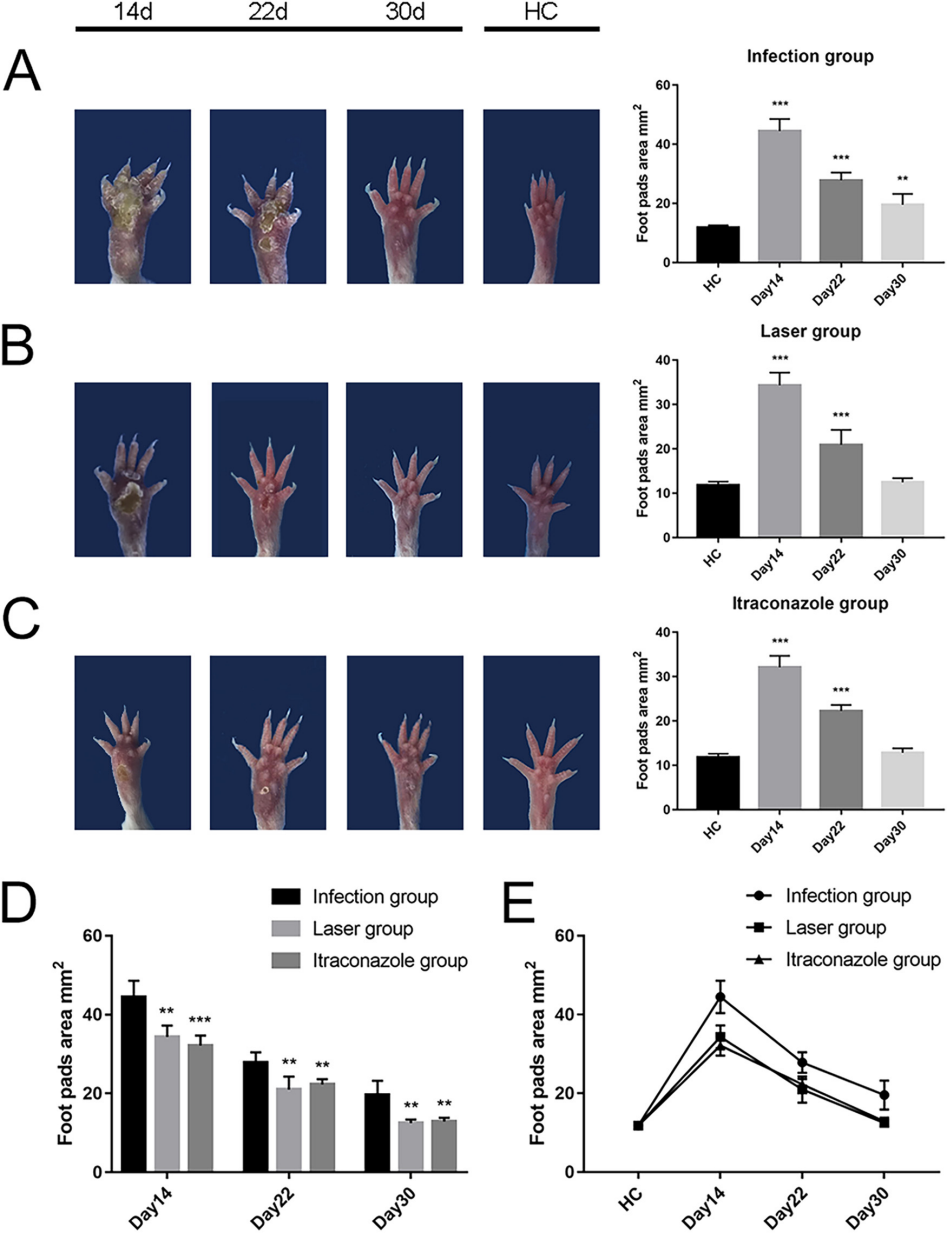
The footpad sizes of S. globosa-infected mice after different treatments. (A to C) The footpad sizes of mice in the infection (A), laser (B), and itraconazole (C) groups compared to the healthy control group, on days 14, 22, and 30. (D) The footpad sizes of the laser group and itraconazole group compared to the infection group on days 14, 22, and 30. (E) Analysis of the trends of the footpad areas of mice in different groups. **, *P* < 0.01; ***, *P* < 0.001.

The mean footpad size was 11.82 ± 0.81 mm^2^ in the healthy control (HC) group. We found that on days 14 and 22, the footpad sizes of mice were significantly larger in all three study groups than in the HC group (*P* < 0.001) (Table 1, Fig. 7A to C). The footpad size of mice in the infection group was significantly larger than those of mice in the laser and itraconazole treatment groups (Fig. 7D). On day 30, the footpad sizes of the laser group and the itraconazole group had returned to nearly normal, a faster recovery than was seen in the infection group (Fig. 7D and E).

**TABLE 1:** The footpad sizes of S. globosa-infected mice

Treatment group	Mean footpad area ± SD (mm^2^) on day^*a*^:		
	14 (*n* = 6)	22 (*n* = 6)	30 (*n* = 6)
Untreated infection	44.48 ± 4.10***	27.81 ± 2.64***	19.55 ± 3.67**
Laser	34.33 ± 2.87***	20.95 ± 3.32***	12.50 ± 0.88
Itraconazole	32.13 ± 2.56***	22.29 ± 1.33***	12.86 ± 0.96

a Statistically significant differences for the mean value compared to that of the healthy control group are shown as follows: **, *P* < 0.01; ***, *P* < 0.001.

### Effects of laser irradiation on mouse histopathology after S. globosa infection.

**FIG 8:**
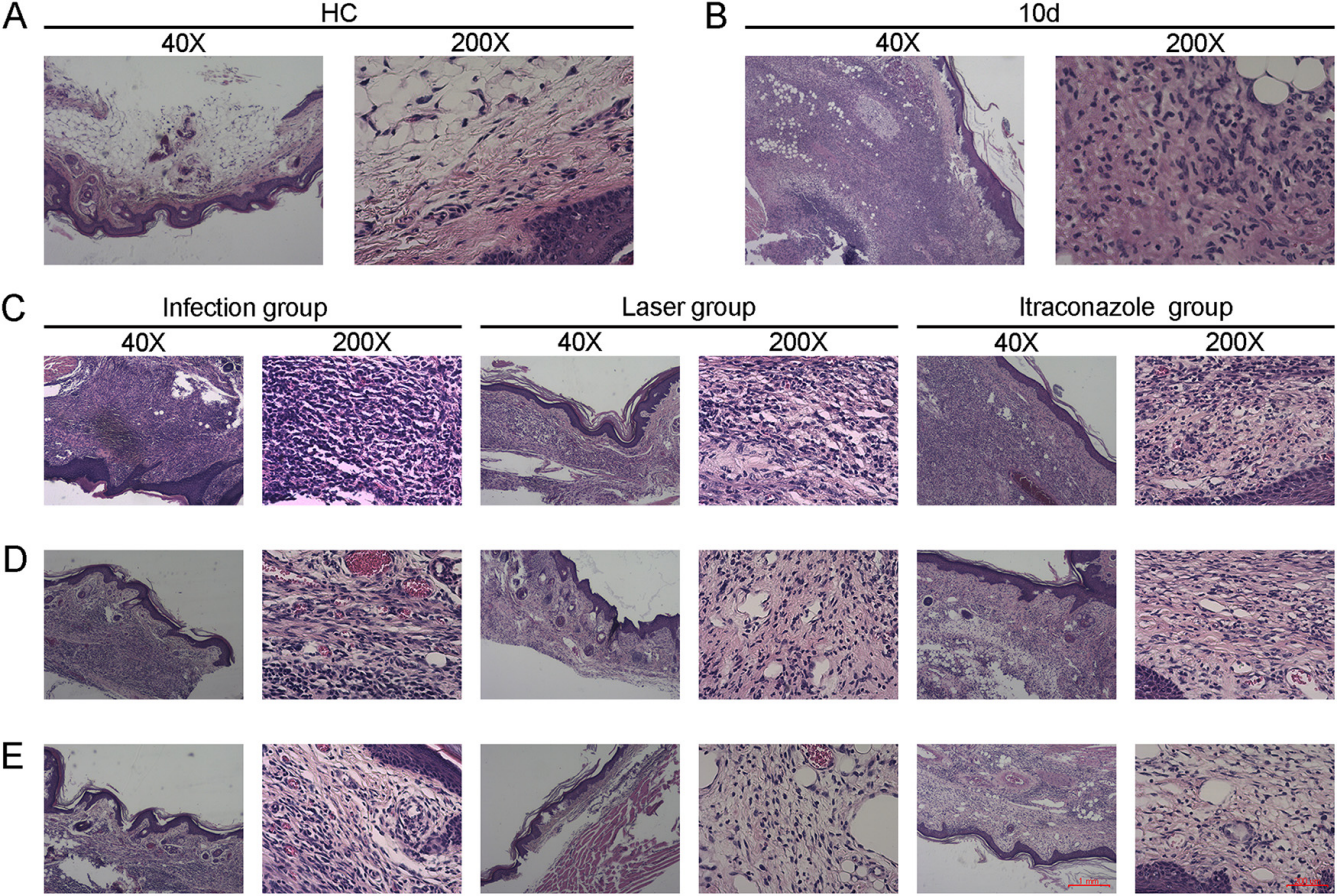
Histopathology of skin tissues of S. globosa-infected mice on days 14, 22, and 30 after different treatments. (A) Healthy control (HC) group. (B) Study group on day 10. (C to E) Histopathological changes of skin tissues of the different treatment groups on days 14 (C), 22 (D), and 30 (E).

The skin histology of HC mice is shown in Fig. 8A. We randomly took the footpad tissues of 3 mice in the study group for histopathology examination on day 10. We observed the formation of a large number of epithelioid cells, lymphocytes, and granulomatous changes, which proved that the mouse model was built successfully (Fig. 8B). After that, we examined the histology of skin in the areas where S. globosa cells had been inoculated once a week.

On day 14, we observed suppurative inflammation with extensive necrosis in all study groups. The inflamed tissue contained many lymphocytes in the outer layer of the lesions (Fig. 8C). On day 22 after infection, the pus-like inflammatory focus was limited or had disappeared in the laser and itraconazole groups. In comparison, we still observed numerous lymphocytes in the infection group (Fig. 8D). On day 30, the skin lesions in all study groups were significantly thinned and the inflammatory cells were significantly reduced. Additionally, we found that the mice in the laser group and the itraconazole group had fewer lymphocytes than the mice in the infection group, while the skin on their footpads was thinner and closer to that of the HC group (Fig. 8E). In brief, the histopathological changes in these groups (Fig. 8) were consistent with the results of the skin infection (Fig. 7).

### Effects of laser irradiation on NLRP3, caspase-1, and caspase-3 expression in mice after S. globosa infection.

**FIG 9:**
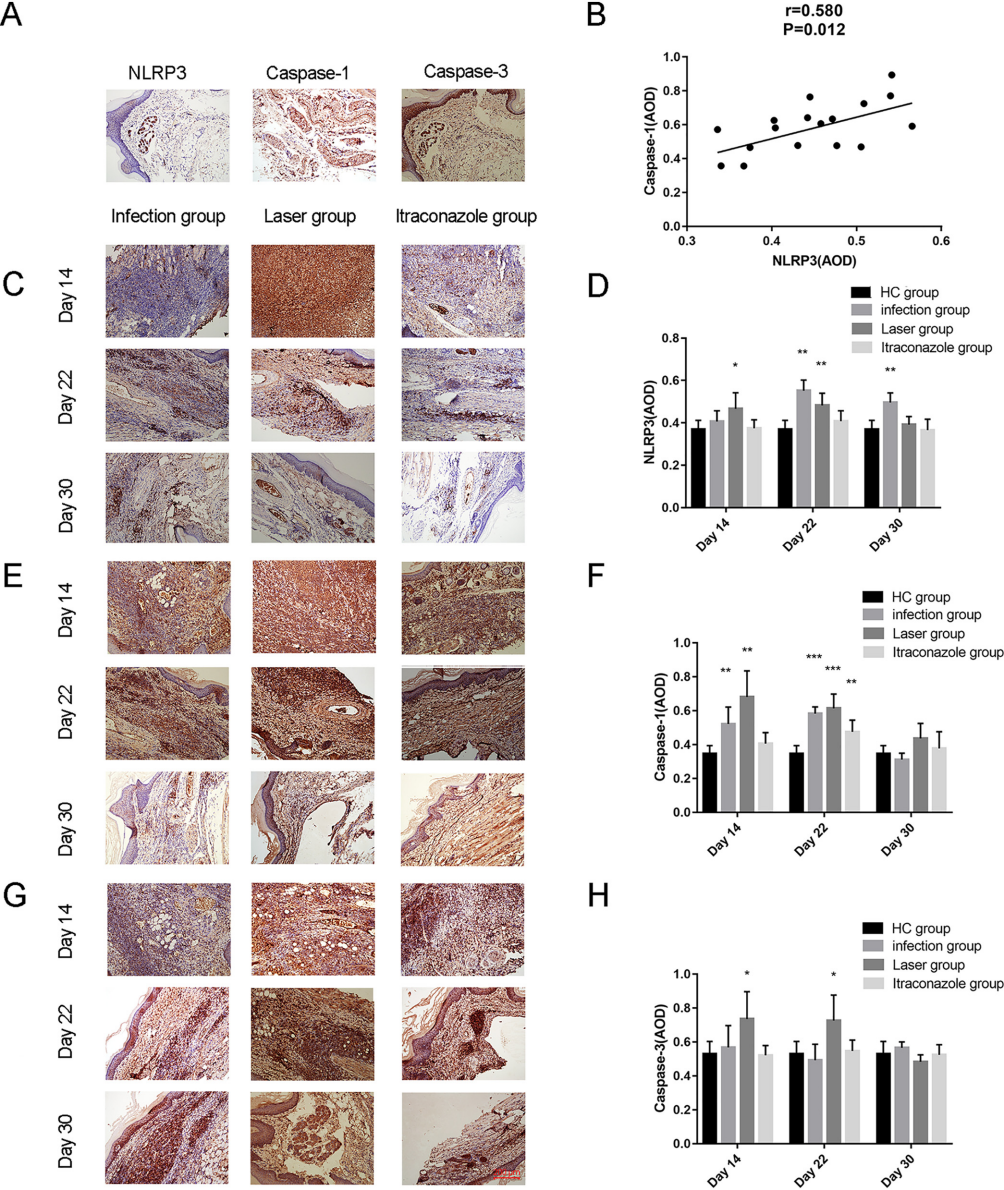
Immunohistochemical (IHC) analysis of NLRP3, caspase-1, and caspase-3 expression levels in samples from S. globosa-infected mice after different treatments. We compared the NLRP3, caspase-1, and caspase-3 levels by measuring the average optical density (AOD) of skin tissues in different treatment groups on days 14, 22, and 30. (A) Representative examples of NLRP3, caspase-1, and caspase-3 expression in tissues of healthy control mice. (B) There was a positive correlation between the levels of expression of NLRP3 and caspase-1 in samples from the laser group. (C and D) The expression of NLRP3 in samples from the infection, itraconazole, and laser groups on days 14, 22, and 30 compared to the expression in samples from the healthy control group. (E and F) The expression of caspase-1 in samples from the infection, itraconazole, and laser groups on days 14, 22, and 30 compared to the expression in samples from the healthy control group. (G and H) The expression of caspase-3 in samples from the infection, itraconazole, and laser groups on day 14, 22, and 30 compared to the expression in samples from the healthy control group. Magnification, ×200. NLRP3, Nod-like receptor thermoprotein domain-related protein 3. *, mean value compared to HC group, *P* < 0.05; **, mean value compared to HC group, *P* < 0.01; ***, mean value compared to HC group, *P* < 0.001.

The expression of NLRP3, caspase-1, and caspase-3 in mouse footpad tissues was detected by immunohistochemistry (IHC), using measurement of average optical density to evaluate the pyroptosis and apoptosis (Table 2 and Fig. 9A). We found that NLRP3 was positively related to the caspase-1 levels (*r* = 0.580, *P* = 0.012) (Fig. 9B) in the laser group. Furthermore, compared to the levels in the HC group (0.37 ± 0.04), the NLRP3 levels were upregulated in the laser group (0.47 ± 0.08) (*P* = 0.025). There was no significant difference in the infection group on day 14 (0.41 ± 0.05) (*P* = 0.262). On day 22, we found that the NLRP3 levels of the laser (0.48 ± 0.06) (*P* = 0.004) and infection (0.55 ± 0.05) (*P* = 0.004) groups were both higher than that of the HC group. On day 30, the NLRP3 level of the infection group (0.50 ± 0.05) (*P* = 0.004) was still higher than that of the HC group, while the level returned to almost normal in the laser group (0.39 ± 0.04) (*P* = 0.337) (Fig. 9C and D). In addition, the changes in caspase-1 levels were almost the same as the changes in NLRP3 levels (Fig. 9E and F). These data suggested that the NLRP3/caspase-1 pyroptosis pathway was activated after laser irradiation. However, compared with the levels in the HC group, no significant differences were found in the itraconazole group, which indicates that the NLRP3/caspase-1 pyroptosis pathway did not participate in the action of itraconazole against S. globosa (Fig. 9C to F).

**TABLE 2:** NLRP3, caspase-1, and caspase-3 expression levels found in AOD assay of tissue samples from S. globosa-infected mice^*a*^

Parameter	Treatment group	Avg optical density ± SD on day:		
		14 (*n* = 6)	22 (*n* = 6)	30 (*n* = 6)
NLRP3	Untreated infection	0.41 ± 0.05	0.55 ± 0.05	0.50 ± 0.05
	Laser	0.47 ± 0.08	0.48 ± 0.06	0.39 ± 0.04
	Itraconazole	0.37 ± 0.04	0.41 ± 0.05	0.37 ± 0.05
Caspase-1	Infection	0.52 ± 0.10	0.58 ± 0.04	0.31 ± 0.04
	Laser	0.68 ± 0.15	0.61 ± 0.08	0.44 ± 0.09
	Itraconazole	0.41 ± 0.06	0.47 ± 0.07	0.38 ± 0.10
Caspase-3	Infection	0.57 ± 0.13	0.49 ± 0.09	0.57 ± 0.03
	Laser	0.74 ± 0.16	0.73 ± 0.15	0.48 ± 0.04
	Itraconazole	0.52 ± 0.06	0.55 ± 0.06	0.52 ± 0.06

a AOD, average optical density.

Caspase-3 is a critical mediator of apoptosis. We measured the expression of caspase-3 in footpad tissues in different groups. Compared with the levels in the HC group (0.53 ± 0.07), our data showed that caspase-3 levels were upregulated in the laser group on days 14 (0.74 ± 0.16) (*P* = 0.014) and 22 (0.73 ± 0.15) (*P* = 0.016), whereas no significant difference was found in the infection and itraconazole groups, indicating that apoptosis was also activated by laser irradiation *in vivo* (Fig. 9G and H).

### Effects of laser irradiation on IL-17, IFN-γ, and TGF-β1 levels after S. globosa infection.

**FIG 10:**
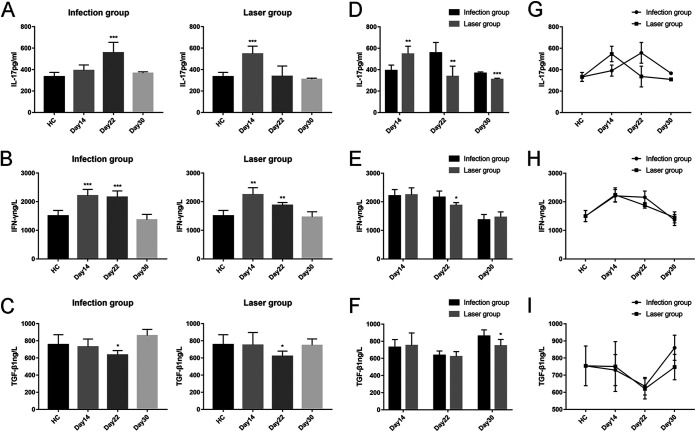
IL-17, IFN-γ, and TGF-β1 levels in peripheral blood samples from S. globosa-infected mice after different treatments. We compared the levels of IL-17, IFN-γ, and TGF-β1 on days 14, 22, and 30. (A to C) The IL-17, IFN-γ, and TGF-β1 levels in mice from the infection and laser groups compared to the levels in mice from the healthy control group. (D to F) The IL-17, IFN-γ, and TGF-β1 levels in mice from the laser group compared to the levels in mice from the infection group. (G to I) Analysis of the trends of the IL-17, IFN-γ, and TGF-β1 levels in both groups. Statistical significance regarding the times of treatment was determined. *, *P* < 0.05; **, *P* < 0.01; ***, *P* < 0.001.

We compared the levels of interleukin 17 (IL-17), interferon gamma (IFN-γ), and transforming growth factor β1 (TGF-β1) in peripheral blood on days 14, 22, and 30 after infection (Table 3 and Fig. 10). The mean IL-17 level of the HC group was 332.08 ± 41.92 pg/ml. Compared to the HC group, we found that the mean IL-17 level of the laser group increased earlier than that of the infection group, on day 14. On day 22, the IL-17 level of the infection group was still higher than that of the HC group, while a significant decrease was observed in the laser group. In addition, on days 22 and 30, we found that the mean IL-17 levels of the laser group were significantly lower than those of the infection group (Fig. 10A, D, and G). These results indicated that laser irradiation may have played an antifungal role by regulating Th17 cell immunity.

**TABLE 3:** Levels of IL-17, IFN-γ, and TGF-β1 in peripheral blood samples from S. globosa-infected mice

Parameter	Treatment group	Mean ± SD or median (range) on day^*a*^:		
		14 (*n* = 6)	22 (*n* = 6)	30 (*n* = 6)
IL-17 (pg/ml)	Untreated infection	391.19 ± 51.49	556.92 ± 96.57***	366.06 ± 12.67
	Laser	546.23 ± 71.96***	336.01 ± 96.89	309.09 ± 10.68
IFN-γ (ng/liter)	Untreated infection	2,208.33 ± 220.25***	2,159.38 ± 217.54***	1,362.50 ± 194.15
	Laser	2,371.88 (2067.19,2395.31)**	1,873.96 ± 96.13**	1,455.56 ± 193.12
TGF-β1 (ng/liter)	Untreated infection	729.84 ± 90.44	635.48 ± 50.90*	859.82 ± 73.11
	Laser	750.54 ± 145.41	640.32 (621.77, 651.61)*	747.32 ± 74.15

a Normally distributed data are shown as the mean values ± standard deviations, and continuous variables are shown as the median values and ranges. Statistically significant differences for the mean value compared to that of the healthy control group are shown as follows: *, *P* < 0.05; **, *P* < 0.01; ***, *P* < 0.001.

The mean IFN-γ level of the HC group was 1,500.00 ± 196.69 ng/liter. Compared to the level in the HC group, we found that the IFN-γ levels of all study groups were raised on days 14 and 22. In addition, on day 22, the mean IFN-γ level of the laser group was significantly lower than that of the infection group (Fig. 10B, E, and H). These results indicated that Th1 cell immunity may also play a part in the activity induced by laser therapy.

The mean TGF-β1 level in the HC group was 754.57 ± 115.64 ng/liter. In our previous study, we found that TGF-β1 was upregulated in the nonacute phase in patients with sporotrichosis, which suggested that it participated in chronic sporotrichosis (20). In this study, no significant change was observed in any of the study groups (Fig. 10C, F, and I). Our analysis indicated that sporotrichosis had been cured before the mobilization of non-acute-phase Treg cell immunity in the laser treatment group.

### Positive correlations between NLRP3 and IL-17 and IFN-γ levels after laser irradiation.

**FIG 11:**
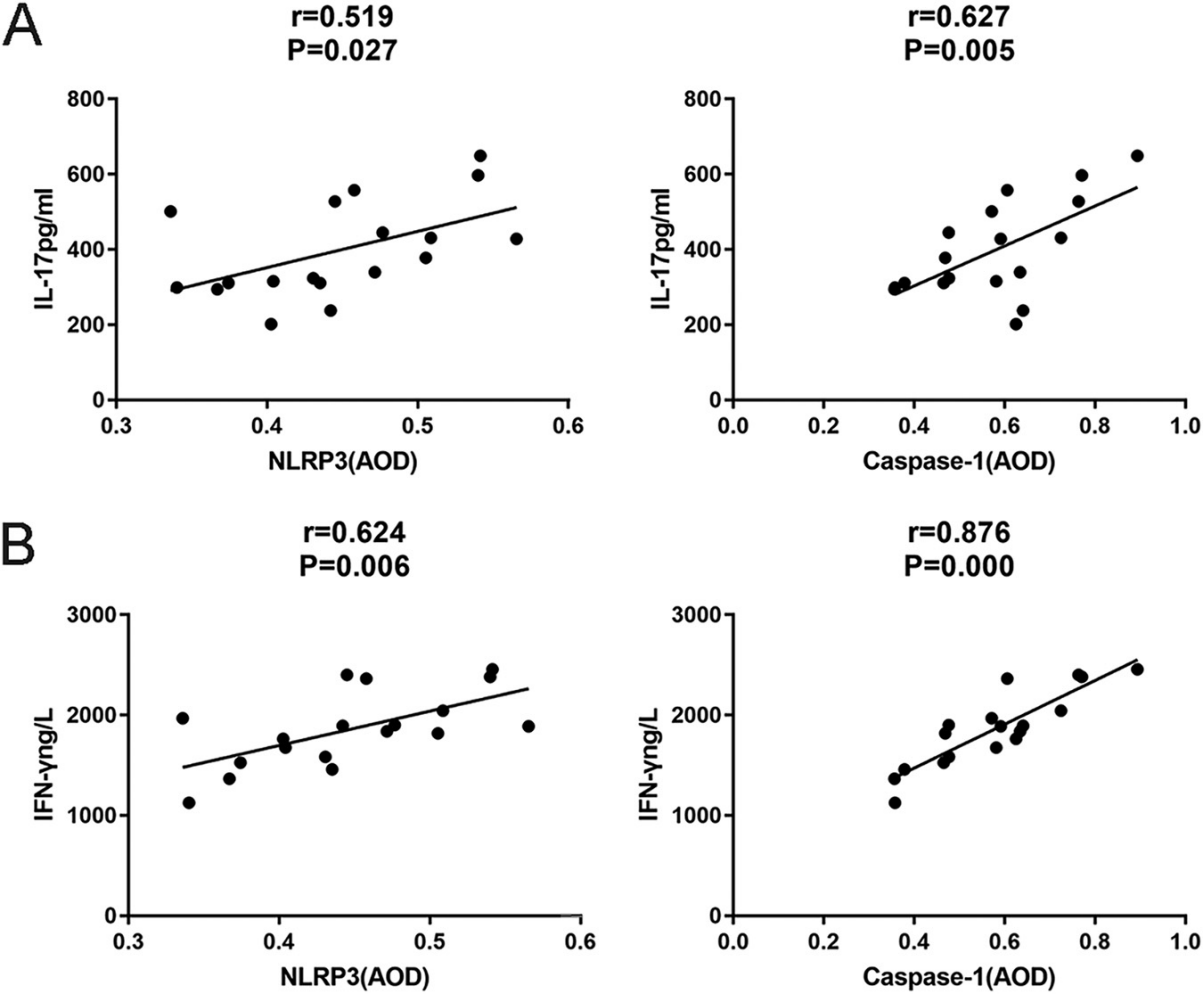
Correlations between NLRP3 and IL-17 and IFN-γ levels in S. globosa-infected mice after laser irradiation. Positive correlations between NLRP3, IL-17, and IFN-γ levels after laser irradiation by IHC. (A) There was a positive correlation between peripheral blood IL-17 levels and NLRP3 and caspase-1 levels in the laser irradiation group. (B) There was a positive correlation between peripheral blood IFN-γ levels and NLRP3 and caspase-1 levels in the laser irradiation group. AOD, average optical density.

In the laser group, we found that the NLRP3 and caspase-1 levels of footpads were positively correlated with the levels of IL-17 (*r* = 0.519, *P* = 0.027, and *r* = 0.627, *P* = 0.005) (Fig. 11A) and IFN-γ (*r* = 0.624, *P* = 0.006, and *r* = 0.876, *P* = 0) (Fig. 11B) in the peripheral blood, indicating that laser irradiation activated Th17 and Th1 cell immunity via the NLRP3/caspase-1 pyroptosis pathway.

## DISCUSSION

Our *in vitro* results showed that laser irradiation (at an energy density of 191 J/cm^2^) was highly effective, inducing morphological changes, ROS accumulation, disruption of the mitochondrial membrane potential, oxidative stress, cell cycle block, and metacaspase activation in S. globosa cells. The outcome suggested that apoptosis was induced as part of the activity of laser therapy against S. globosa cells. In addition, caspase-3 (a critical mediator of apoptosis) was upregulated after laser irradiation of the footpads of mice. After laser treatment, the skin lesions and histopathological changes were cured as fast as after itraconazole treatment. Moreover, we found that NLRP3, caspase-1, IL-17, and IFN-γ were upregulated in the early phase in the footpads of mice that received the laser treatment, and they had a positive relationship. This result suggests that the laser therapy may regulate Th1 and Th17 cell immunity by activating the NLRP3/caspase-1 pyroptosis pathway in the antifungal therapy process. All of these results indicate that Nd:YAG 1,064-nm laser therapy can be used against S. globosa infection. The specific mechanism is activation of the apoptosis and NLRP3/caspase-1 pyroptosis pathway.

Currently, sporotrichosis is mainly treated with antifungal drugs, photodynamic therapy, hyperthermia, cryotherapy, and surgery. Among these, antifungal treatment with itraconazole is the most common protocol, but the drawbacks include a long period of drug administration, poor patient compliance, a variety of adverse effects, and drug resistance (4–6). Therefore, it is urgent to explore new treatment methods. Nd:YAG 1,064-nm laser therapy is a promising addition to the traditional antifungal therapies.

After laser treatment, the scanning electron microscopy (SEM) experiment showed significant shrinkage of the S. globosa cell surface and a large amount of leakage of cell contents. Moreover, using Hoechst/propidium iodide double staining, we further found that Nd:YAG 1,064-nm laser treatment caused apoptosis and necrosis of S. globosa cells. Thus, we speculated that apoptosis is one of the major mechanisms of laser therapy against S. globosa. To explore its specific mechanism, many characteristics typical of the process of apoptosis were tested. Among them, abnormality of the mitochondrial membrane potential is one of the earliest events (23). In this study, we found that the mitochondrial activity of S. globosa was disrupted after laser irradiation. Mitochondria are the control center of cellular life activity and the regulation center of apoptosis. If the mitochondria are damaged, abnormal leakage of electrons in the mitochondrial respiratory chain will occur, with the accumulation of ROS in the mitochondria (24).

ROS are the major mediators of fungal apoptosis, causing cell death by oxidizing biological macromolecules (25). Early studies showed that the attack on mitochondria by ROS overproduction could inhibit the mitochondrial respiratory function, leading to fungal cell death (26–28). Those reports coincide with our results, which showed that the level of ROS in S. globosa was significantly increased after laser treatment. In addition, excessive production of ROS causes oxidative stress, resulting in serious damage to lipids, proteins, and other biological structures. It also promotes cell apoptosis. H_2_O_2_ can destroy fungal DNA and protein, while SDS damages the cell wall. In this study, we found that laser irradiation increased the sensitivity of S. globosa to H_2_O_2_ or SDS, resulting in inhibition of the growth of S. globosa.

As we all know, the cell cycle is the basic process of cellular life activity. Cellular apoptosis induces increased ROS, which causes certain damage to the DNA and then affects the cell cycle process (29). In this study, we found that the cell cycle of S. globosa is arrested in the S phase after laser treatment, which blocks cell division. The causes may be incomplete DNA repair and decreased replication as the cells enter the G_2_/M phase from the S phase. Moreover, the upregulation of transcription factors like c-Myc, c-Jun, and c-Fos or changes in cyclin and cyclin-dependent kinase accelerate the G_1_/S transformation, leading to more cells entering the S phase.

Madeo et al. (30) found that both caspase-dependent and caspase-independent apoptotic pathways participate in fungal cell apoptosis. The early studies showed that ROS accumulation enhanced the metacaspase activity inside the fungus, resulting in fungal apoptosis (31–33). In this study, we further investigated the changes in the metacaspase activity of S. globosa cells after laser treatment. The results showed that metacaspase was activated by the Nd:YAG 1,064-nm laser, whereas metacaspase is inactive under normal conditions, indicating that the laser activates the caspase-dependent pathway in S. globosa apoptosis. Moreover, caspase-3 is a critical mediator of apoptosis, and we explored its expression changes *in vivo* by IHC. The results showed that the caspase-3 levels were upregulated by the laser treatment and that apoptosis was activated by laser irradiation. Our earlier study showed that Th1, Th17, and Treg cells participate in the progression of sporotrichosis. The levels of IL-17 and IFN-γ are upregulated in acute disease and downregulated in nonacute disease, which means that CD4^+^ T cells participate in the body’s resistance to sporotrichosis (20). Guan et al. (34) found that a fractional CO_2_ laser could be used for treating onychomycosis by driving stronger local Th1 responses due to an imbalance of IFN-γ/IL-4 expression. de Castro et al. (35) reported that photobiomodulation induces a proinflammatory Th1-driven response against Candida albicans in monocytes and macrophages. Zhang et al. (36) showed that a 308-nm excimer laser is effective in reducing the infiltration of CD4^+^ T cells into the skin lesions of vitiligo patients, while it promotes the infiltration of Treg cells that secrete TGF-β and IL-10, especially in the stable stage. The study indicates that the laser treatment affects the immune response. Moreover, Baroni et al. (10) found that Nd:YAG 1,064-nm laser treatment increases TGF-β expression in the treatment of Candida albicans infection. The results described above indicate that various types of lasers play antifungal roles by regulating CD4^+^ T cell immunity. However, whether the Nd:YAG 1,064-nm laser can regulate immunity in sporotrichosis is not yet clear. In this study, we found that the levels of IL-17 and IFN-γ in S. globosa-infected mice changed significantly after Nd:YAG 1,064-nm laser treatment, while the levels and speed of the changes drove faster recovery than was seen in the untreated infected mice. We speculate that laser treatment activated Th1 and Th17 responses that promoted antifungal immunity.

The NLRP3 inflammasome is composed of NLRP3, apoptosis-related spot-like protein (ASC), and caspase-1. It can recognize danger signals and activate body immunity. Zhao et al. (21) showed that adjusting the peroxisome proliferator-activated receptor gamma (PPAR-γ)/HMGB1/NLRP3 axis can inhibit inflammation and apoptosis in myocardial injury. Frank et al. (37) found that caspase-1^−/−^ and ASC^−/−^ mice display diminished Th1/Th17 responses, followed by increased fungal outgrowth and lower rates of survival. This indicates that caspase-1 and ASC have strongly protective antifungal capacities through controlling Th1 and Th17 responses during disseminated candidiasis, due to activation of the NLRP3/ASC/caspase-1 pyroptosis pathway. In this process, caspase-1 cleaves IL-1β and IL-18 into active proteins, whereupon IL-1β is important for inducing Th17 responses and IL-18 drives Th1 responses. In a word, these cytokines are activated upon the initiation of the adaptive T-helper responses.

Moreover, our previous study showed that S. globosa infection increased Treg cell levels in nonacute patients (20). However, in this study, no significant increase in Treg cell levels was observed in the laser treatment group at any time, which may be due to the fact that the sporotrichosis had been cured before the mobilization of non-acute-phase Treg cell immune activity. Some previous studies showed that NLRP3 is also associated with apoptosis. However, our study did not find a correlation between the NLRP3 and caspase-3 levels, suggesting that laser-regulated apoptosis and pyroptosis are two unrelated pathways.

The advantages of Nd:YAG 1,064-nm laser treatment include high efficiency, low cost, safety, lack of drug resistance, etc. The Nd:YAG 1,064-nm laser can be used as an alternative therapy for the elderly, children, pregnant women, or patients with drug-resistant infections who are not suitable for conventional medical treatment. The melanin in the S. globosa cell wall can selectively absorb the Nd:YAG 1,064-nm laser beam, which produces a photothermal effect and avoids damage to the surrounding tissues. Combining the Nd:YAG 1,064-nm laser with antifungal drugs increased the effect of the sporotrichosis treatment and was better than using antifungal drugs alone. In this process, the laser treatment shortened the course of sporotrichosis, reduced the side effects of azole antifungal drugs, and did not increase the generation of scars (14). In this study, we further proved that the Nd:YAG 1,064-nm laser treatment could also work through the mechanisms of apoptosis and pyroptosis, indicating that it could exert anti-S. globosa effects through a variety of mechanisms.

Our study has limitations. An NLRP3 or caspase-1 inhibitor weakens the antifungal effect of the Nd:YAG 1,064-nm laser, which needs study in the future. A larger sample will improve the reliability of the statistical analysis. In addition, further studies on larger samples are needed, and the long-term effect of laser treatment needs further observation.

### Conclusions.

Our data provided evidence that Nd:YAG 1,064-nm laser treatment could effectively inhibit the growth of S. globosa *in vitro* by activating fungal apoptosis. In this process, significant changes were observed in the morphological structure, ROS accumulation, mitochondrial membrane potential, oxidative stress, cell cycle progression, and metacaspase activation in S. globosa cells. In addition, caspase-3, which is a critical effector of apoptosis, was also upregulated after laser irradiation *in vivo*. More importantly, we further revealed that after laser therapy, the NLRP3/caspase-1 pyroptosis pathway was also activated to promote Th1 and Th17 cell immunity against S. globosa infection in mice. At the same time, its effect was almost equivalent to that of itraconazole, which is often used in clinical treatment of sporotrichosis. In conclusion, our findings support that Nd:YAG 1,064-nm laser treatment may be a safe and effective new type of treatment for clinical sporotrichosis.

## MATERIALS AND METHODS

### Preparation of S. globosa.

We used S. globosa cells from the strain preserved in our laboratory. The S. globosa was confirmed by both mycelial-to-yeast phase conversion culture and molecular identification. Briefly, the isolates were grown on Sabouraud’s dextrose agar for 7 days. Then, the fungal colonies were transferred to brain heart infusion broth and incubated in a rotary shaker (150 rpm) at 37°C for 7 days. After centrifugation, the culture suspension was resuspended in phosphate-buffered saline (PBS) and diluted to 1 × 10^8^ cells/ml using a hemocytometer. Using the dilution equation, 2-μl aliquots of each dilution were inoculated onto brain heart infusion agar plates and incubated for 7 days before Nd:YAG 1,064-nm laser irradiation.

### S. globosa viability assay.

We used a cell counting kit-8 (CCK-8) viability assay (Beyotime Biotechnology) to compare the laser’s antifungal effects at 200 J/cm^2^, 400 J/cm^2^, and 600 J/cm^2^. Samples (100-μl/well suspensions) from the different groups were placed into 96-well microplates, and 10 μl of the CCK-8 cell viability detection reagent was added at the same time. After 2 h of incubation, the absorbance at 450 nm was measured with a microplate reader.

### *In vitro* Nd:YAG 1,064-nm laser irradiation.

A total of 93 colonies were randomly and blindly allocated to the control group (*n* = 49) and the study group (*n* = 44). The colonies of the control group were not treated. The colonies of the study group were irradiated vertically using a long-pulsed Nd:YAG 1,064-nm laser (Jilin Provincial King Laser Technology Company, Ltd. Changchun, China) with an energy dose of 400 J/cm^2^.

Due to the strong penetrability of the 1,064-nm laser beam, most of the energy emitted by the laser passed through the culture dish and was lost. We used an OPHIR laser power meter (1000W-LP2-34) to measure the energy actually absorbed by S. globosa cells and calculated the energy density. The actual energy density absorbed by S. globosa cells was calculated as follows: actual energy density = *b*/π *r*^2^, where *b* is the actual energy absorbed by S. globosa cells and *r* is the spot semidiameter of the laser beam.

When we used an energy of 400 J/cm^2^, the spot diameter of the laser beam was 4 mm, with the laser emitting 30-ms pulses at a 1-Hz pulse/frequency, and the actual energy density received by S. globosa cells was 191 J/cm^2^. During laser irradiation, we positioned the laser head 5 mm from the plate. The laser beam irradiated the entire colony and moved in a spiral shape from the periphery to the center. Each colony was irradiated with 8 to 10 light spots. After the entire colony was irradiated, a 2-min pause was taken. Then, the treatment and pause were repeated three times.

The culture dishes of both groups were put into a 37°C incubator to continue the cultivation. The relevant experiments were performed 12 h later. The yeasts obtained from the cultures were suspended in sterile distilled water and diluted to 2 × 10^7^ CFU/ml, as determined with a hemocytometer.

### SEM analysis.

The yeasts of both groups were cultured in 24-well tissue culture plates with cell slides for 4 h. We discarded the supernatant and washed each well with PBS once, and then fixed the cells with 2.5% glutaraldehyde (0.2 M phosphate buffer, pH 6.0) at 4°C for 1 h. After the cells were fixed, the disks were rinsed 3 times with PBS and dehydrated in a graded ethanol series (30%, 50%, 70%, 80%, and 90% for 7 min each, and then 100% for 10 min). The disks were subjected to critical point drying prior to being coated by gold sputter and observed using a SEM (Hitachi, Tokyo, Japan).

### Cell apoptosis and necrosis.

In order to evaluate the effect of laser treatment on S. globosa necrosis and apoptosis, the cells of the study and control groups were centrifuged at 5,000 × *g* for 5 min and resuspended in 800 μl staining buffer. We added 5 μl of Hoechst 33342 and 5 μl of propidium iodide (Beyotime Biotechnology) to the suspension and mixed them in by inversion. The suspension was ice bathed for 30 min and washed once with PBS. Fungal smears were observed under a confocal laser scanning microscope (Zeiss LSM 780, German).

### Stress sensitivity test.

The S. globosa cells of both groups were collected by centrifugation and diluted 2 times using a 10× *g*radient. Then, 5 μl of each dilution was spotted onto yeast extract-peptone-glycerol (YPG) solid medium containing SDS and H_2_O_2_, and the plates inverted and placed at 37°C for 7 days until colonies had formed.

### Measurement of mitochondrial membrane potential.

The laser-treated cells (1 × 10^8^ CFU/ml) were washed and then resuspended in PBS (pH 7.4). Then, JC-1 dye (tetraethylbenzimidazolylcarbocyanine iodide; Beyotime Biotechnology) was added to a 5 μM final concentration. The mixture was incubated at 37°C for 20 min. The fluorescence intensity was recorded with a microplate fluorescence reader at an excitation wavelength of 490 nm and emission wavelength of 530 nm.

### ROS accumulation.

We diluted 2′,7′-dichlorofluorescein diacetate (DCFH-DA) (Beyotime Biotechnology) with yeast extract-peptone-dextrose (YPD) liquid medium to 1:1,000 for a final concentration of 10 μM.

After one night of incubation, the S. globosa cells were collected and suspended in diluted DCFH-DA (final concentration, 10^8^ cells/ml) in a 37°C incubator for 20 min. The cells were washed three times with YPD liquid medium and directly stimulated with the active oxygen positive solvent. After 20 min, the fluorescence density (excitation at 488 nm and emission at 525 nm) was measured using a fluorescence reader (Molecular Devices, USA). The formula for calculating the ROS accumulation level was (*F*_text_ − *F*_blank_)/(*F*_control_ − *F*_blank_), where *F*_text_ was the fluorescence value of the sample after laser treatment, *F*_control_ was the fluorescence value of the untreated sample, and *F*_blank_ was the fluorescence value of the cell-free well.

### Cell cycle analysis.

The S. globosa cells of both groups were centrifuged at about 1,000 × *g* for 5 min to precipitate the cells. Subsequently, the cells were resuspended in 1 ml of ice bath-prechilled PBS and transferred to a 1.5-ml centrifuge tube. The cells were then added to 1 ml of ice bath-prechilled 70% ethanol, fixed at 4°C overnight, and centrifuged at 1,000 × *g* for 5 min to precipitate the cells. Next, each pellet was slowly and fully resuspended after the addition of 0.5 ml of propidium iodide staining solution (Beyotime Biotechnology) and incubated at 37°C in the dark for 30 min. Then, all the cell samples were stored in an ice bath away from the light. Using flow cytometry (LSR II; BD, USA), we detected red fluorescence at an excitation wavelength of 488 nm while detecting light scattering. DNA content analysis and light-scattering analysis were performed using the software Modfit LT 3.0.

### Detection of metacaspase activation.

The S. globosa cells of both groups were centrifuged at about 2,000 × *g* for 3 min and resuspended, and then 10 μM CaspACE FITC-VAD-FMK (Promega, Fitchburg, WI, USA) was added and the cells stained at 37°C for 20 min. After this period, the cells were washed twice with PBS and smears examined under a confocal laser scanning microscope (LSM 780; Zeiss, Germany). Meanwhile, the percentage of the cells with activated caspase was detected by measuring the fluorescence absorption value using a fluorescence reader (Molecular Devices, USA). The excitation light wavelength was set at 494 nm, and the emission light wavelength at 518 nm.

### Animals.

A total of 63 female BALB/c mice (age, 8 to 10 weeks; body weight, 16 to 20 g) were purchased from Changchun Yisi Experimental Animal Technology Co., Jilin, China. The animal protocol was approved by the Institutional Ethics Committee for Animal Use in Research, and our animal care followed the animal care guidelines of Jilin University. The ethics document is approved by Jilin University College of Basic Medical Sciences, with ethics number no. 124 in 2021. The mice were housed in groups of 6 animals per cage. We maintained the mice under a 12-h light and 12-h dark cycle with free access to food and water. The mice were allocated to the study group (*n* = 57) and the HC group (*n* = 6).

### Inoculation and antifungal treatments.

After routine sterilization, 0.05-ml amounts of an S. globosa suspension (1 × 10^8^ CFU/ml) were injected into the footpads of mice in the study groups. The skin condition was observed daily. On day 10, we confirmed via histopathology that the mouse models were building successfully. Then, the mice of the study group were divided into an untreated-infection group (*n* = 18), an itraconazole treatment group (*n* = 18), and a laser treatment group (*n* = 18). The antifungal treatments were given. The mice of the itraconazole group were administered itraconazole at a dose of 60 mg/kg of body weight every day by gavage. In the laser group, the injected feet were subjected to long-pulsed Nd:YAG 1,064-nm laser irradiation (energy density, 100 J/cm^2^; spot diameter, 4 mm; pulse duration, 30 ms; pulse frequency, 1 Hz) at room temperature. The laser treatment was administered once every week. After inoculation, the footpads were measured every day using Photoshop CC 2019. Every week, 6 mice from each group were sacrificed, and blood and foot tissue samples were taken to test the serum levels of IFN-γ, IL-17, and TGF-β1 and perform skin histology and immunohistochemistry assays.

### Skin histology.

The skin lesions were isolated and tissue samples fixed with 10% buffered formalin for at least 24 h. The samples were then dehydrated, embedded in paraffin, and sliced into 5-μm sections. For analyzing skin inflammation, the sections were stained with hematoxylin and eosin (HE).

### IHC.

IHC was applied to determine the levels of apoptosis and pyroptosis by detecting the expression of NLRP3, caspase-1, and caspase-3 in paraffin sections of footpad tissues of mice. Briefly, the paraffin-embedded sections were dewaxed and gradually rehydrated before being immersed in an EDTA solution (pH 9.0). After the paraffin-embedded sections were heated at 120°C for 150 s, the sections was slowly cooled to room temperature. After fixation, the sections were blocked with immunostain blocking solution and incubated with primary antibody at 4°C overnight. The sections were treated with the following primary antibodies (Beyotime Institute of Biotechnology, Jiangsu, China): rabbit NLRP3 monoclonal antibody (AF2155, 1:100), rabbit caspase-3 polyclonal antibody (AF0081, 1:100), and rabbit caspase-1 monoclonal antibody (AF1681, 1:400). After washing with PBS, the sections were incubated with horseradish peroxidase (HRP)-conjugated secondary antibodies for 20 min at 37°C, visualized with diaminobenzidine, and counterstained with hematoxylin, followed by treatment with hydrochloric acid ethanol for differentiation, phosphate buffer solution for returned to blue an ethanol gradient for dehydration, xylene for transparency, and neutral gum for sealing.

### Enzyme-linked immunosorbent assay.

Peripheral blood mononuclear cells were separated from whole blood samples drawn from the sacrificed BALB/c mice using Ficoll density gradient centrifugation. The serum levels of IFN-γ, IL-17, and TGF-β1 were measured with enzyme-linked immunosorbent assay (ELISA) kits (mlbio, Shanghai, China) according to the manufacturer’s instructions. The absorbance at 450 nm was measured using a 96-well plate reader.

### Statistical analysis.

Statistical analyses were conducted using SPSS version 24 (SPSS, Chicago, IL, USA). Data were summarized as the median values and ranges for nonnormally distributed data or mean values ± standard deviations for normally distributed data. The quantitative analysis of NLRP3, caspase-1, and caspase-3 was performed by assessing the average optical density using the Image J software. One-way analysis of variance (ANOVA), the Kruskal-Wallis H test, and the Mann-Whitney U test were performed to evaluate between-group differences. Statistical significance was set at a *P* value of <0.05.
